# Ultrafast growth of large single crystals of monolayer WS_2_ and WSe_2_

**DOI:** 10.1093/nsr/nwz223

**Published:** 2020-01-08

**Authors:** Zhengwei Zhang, Peng Chen, Xiangdong Yang, Yuan Liu, Huifang Ma, Jia Li, Bei Zhao, Jun Luo, Xidong Duan, Xiangfeng Duan

**Affiliations:** 1 State Key Laboratory for Chemo/Biosensing and Chemometrics, College of Chemistry and Chemical Engineering, Hunan University, Changsha 410082, China; 2 Department of Applied Physics, School of Physics and Electronics, Hunan University, Changsha 410082, China; 3 Center for Electron Microscopy, Institute for New Energy Materials and Low-Carbon Technologies, School of Materials, Tianjin University of Technology, Tianjin 300384, China; 4 Department of Chemistry and Biochemistry, University of California, Los Angeles, CA 90095, USA

**Keywords:** monolayer transition metal dichalcogenides, large single crystal, CVD, ultrafast growth

## Abstract

Monolayer transition metal dichalcogenides (TMDs) have attracted considerable attention as atomically thin semiconductors for the ultimate transistor scaling. For practical applications in integrated electronics, large monolayer single crystals are essential for ensuring consistent electronic properties and high device yield. The TMDs available today are generally obtained by mechanical exfoliation or chemical vapor deposition (CVD) growth, but are often of mixed layer thickness, limited single crystal domain size or have very slow growth rate. Scalable and rapid growth of large single crystals of monolayer TMDs requires maximization of lateral growth rate while completely suppressing the vertical growth, which represents a fundamental synthetic challenge and has motivated considerable efforts. Herein we report a modified CVD approach with controllable reverse flow for rapid growth of large domain single crystals of monolayer TMDs. With the use of reverse flow to precisely control the chemical vapor supply in the thermal CVD process, we can effectively prevent undesired nucleation before reaching optimum growth temperature and enable rapid nucleation and growth of monolayer TMD single crystals at a high temperature that is difficult to attain with use of a typical thermal CVD process. We show that monolayer single crystals of 450 μm lateral size can be prepared in 10 s, with the highest lateral growth rate up to 45 μm/s. Electronic characterization shows that the resulting monolayer WSe_2_ material exhibits excellent electronic properties with carrier mobility up to 90 cm^2^ V^−1^ s^−1^, comparable to that of the best exfoliated monolayers. Our study provides a robust pathway for rapid growth of high-quality TMD single crystals.

## Teaser

With a reverse flow to control the chemical vapour supply in CVD process, we can effectively prevent the undesired nucleation before reaching optimum growth temperature and enable rapid growth of monolayer TMD single crystals.

## INTRODUCTION

Two-dimensional (2D) transition metal dichalcogenides (TMDs) have attracted considerable attention for their distinct physical properties, such as atomically thin geometry, extraordinary mechanical flexibility, layer-number dependent electronic and optoelectronic properties, and tunable spin and valley polarization [1–4], and their potential applications in 2D electronics, optoelectronics and spintronics [5–14]. However, the studies to date are largely limited to mechanically exfoliated materials with limited yield and size. Scalable growth of large area monolayer TMDs is indispensable for practical applications, and has motivated considerable efforts in developing synthetic strategies to high-quality large area TMD monolayers or few layers, including solid-source chemical vapor deposition (CVD) [9,15–38], and gas source metal-organic CVD [39,40]. Of note, yielding much larger crystal size and incurring less contamination by other unwanted elements (necessary in organometallic precursors), the materials produced by solid-source CVD are often of much higher electronic quality. Recently, wafer-scale growth of 2D crystals (polycrystalline) has also been demonstrated with the use of a solid-source CVD approach [41,42]. Despite significant advancements, the materials produced to date are often of mixed layer thickness, limited single crystal domain size or have slow growth rate, limiting the ability to ensure growth of exclusive monolayer materials over large lateral dimension.

For practical applications of these 2D materials in electronic and optoelectronic devices, growth of large single crystal domains with no grain boundaries is essential for ensuring consistent electronic properties and high device yield. To this end, it is essential to reduce nucleation density in the CVD process [43,44]. This is often achieved by keeping the vapor phase source supply sufficiently low to minimize the probability of nucleation of new crystals and prevent multi-layer formation; however, this markedly slows the lateral growth rate (typically ∼1 μm/s or less) [20,31,34]. Large domain monolayer single crystals are usually achieved by extending the growth time, and it often takes hours or even longer to achieve millimeter-scale monolayer crystals [31,43]. Maximizing the lateral growth rate while minimizing the vertical growth for efficient production of large domain monolayer single crystals represents a critical challenge in 2D crystal growth, and is a topic of considerable fundamental and practical significance [45–48]. With use of specifically designed or catalyzed CVD processes, the lateral growth rate of monolayer graphene can be greatly boosted up to 200 μm/s [44].

Rapid growth of monolayer TMD single crystals is more complicated because of difficulties in controlling vapor phase reactants from the vaporization of solid-source materials or organometallics (vs. the simple methane gas for graphene), and has been insufficiently explored to date. In general, rapid growth of large single crystals requires sufficient precursor supply and a sufficient surface migration rate for the precursors, so that once a monolayer nucleus forms, the precursors can rapidly diffuse to and attach onto the growing crystal before new nuclei form. In this case, a higher temperature is usually desired to ensure sufficient source supply and high surface migration rate. However, in a typical thermal CVD process with solid sources, the higher temperature usually leads to rapid increase of source evaporation and unintentional supply of vapor phase reactant before reaching the targeted growth temperature, resulting in ill-controlled nucleation and growth during the temperature ramping stage to produce highly heterogeneous thin films.

Herein, we employ a reverse flow reactor to ensure highly controlled nucleation and growth at high temperature and enable ultrafast growth of millimeter-scale monolayer TMD single crystals. Specifically, we use the reverse gas flow from the substrate to the source during the temperature ramping stage to prevent the unintended supply of the chemical vapor phase reactant during the temperature ramping stage (Supplementary Fig. S1) [49]. This approach can effectively prevent uncontrolled nucleation and growth before reaching optimum growth temperature, making it possible to reach a higher growth temperature to ensure a sufficient vapor phase reactant supply and sufficient surface migration rate for the rapid growth of large single crystals.

## RESULTS

**Figure 1.**
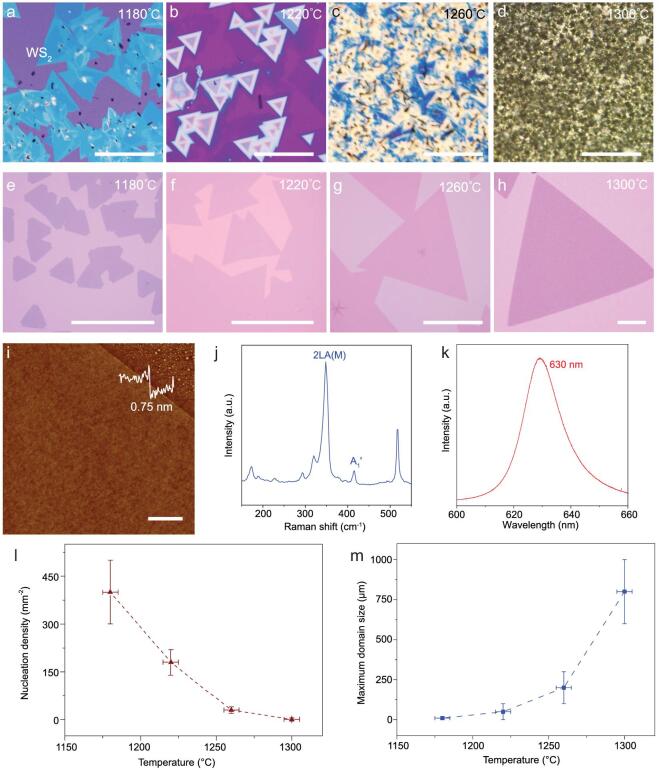
Growth of monolayer WS_2_ single crystals. (a–h) Optical microscope (OM) images of WS_2_ growth with the use of a conventional CVD process (a–d) and a modified CVD process with reverse flow (e–h) kept at different temperatures for 10 s. (i) AFM image and thickness of the monolayer WS_2_ single crystal. (j, k) Raman and photoluminescence spectra of the monolayer WS_2_ single crystal. (l, m) The average nucleation density vs. growth temperature (l) and the maximum domain size vs. the growth temperature (m). Scale bars: 100 μm (a–g), 200 μm (h) and 1 μm (i). Note that we focus on the largest domain here for evaluating the growth rate as smaller domains can also occur where new nuclei form during the middle of the growth period.

Although monolayer TMDs have been successfully produced under precisely controlled conditions with use of a typical thermal CVD process, synthetic conditions are usually highly sensitive [19,21,50]. In this process, the precursor vapor supply is generated by thermal evaporation of solid source and carried downstream to initiate nucleation and growth of 2D crystals on the growth substrate. Here the chemical vapor source is continuously generated and unintentionally supplied to the growth substrate during the temperature ramping stage. Such unintentional supply of the chemical vapor source leads to undesired nucleation and growth before reaching the optimum growth temperature. This makes it particularly difficult to explore the use of a high growth temperature (which is desired for rapid growth of large single crystals), because the higher the designated growth temperature, the greater the unintended chemical vapor supply before reaching the designated growth temperature, which leads to highly heterogeneous thin film deposition with poor control of the thickness and domain size (Fig. 1a–d). For this reason, to minimize excessive vapor supply and undesired material deposition during the temperature ramping stage, growth is typically carried out at a lower temperature (e.g., <1200°C for the source temperature) with a rather low lateral growth rate (∼1 μm/s or less). To break this critical limit, we employed reverse flow during the temperature ramping stage to minimize the undesired supply of gas phase reactants to the growth substrate before reaching the optimum growth temperature, thus enabling rapid growth of large size monolayer single crystals (Fig. 1e, f). An atomic force microscope (AFM) image of the sample shows that the thickness of the resulting WS_2_ single crystal is around 0.75 nm (line scan in Fig. 1i), indicating successful growth of monolayer WS_2_. A typical Raman spectrum of the WS_2_ sample exhibits two prominent peaks at 350 cm^−1^ and 419 cm^−1^ corresponding to the 2LA(M) and A_1_^′^ resonance modes of monolayer WS_2_ (Fig. 1b) [51]. The photoluminescence (PL) spectrum of the sample (Fig. 1c) shows a single peak at 630 nm, also consistent with direct bandgap emission of monolayer WS_2_.

After minimizing the unintentional supply at the temperature ramping stage, we found that nucleation density decreases rapidly with increasing growth temperature (Fig. 1l), which is essential for achieving large single crystal domains and consistent with previous studies on graphene and TMD growth [43,52]. At the same time, the monolayer domain size increases rapidly with the increased growth temperature (Fig. 1m). The maximum domain size reached 800 μm at 1300°C. Note that the temperature we present is the temperature of the source materials. In our single-temperature-zone setup, the substrate temperature is generally lower than the source temperature (Supplementary Table S2).

**Figure 2.**
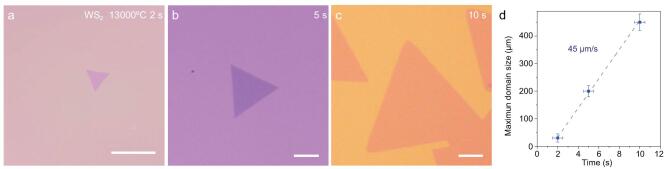
Determination of the maximum growth rate. (a–c) OM images of WS_2_ synthesized at *t* = 2 s (a), 5 s (b) and 10 s (c), respectively. (d) Plot and fit of the maximum domain size of WS_2_ as a function of growth time. The slope reveals an ultrafast growth rate of ∼45 μm s^−1^. All scale bars are 100 μm.

We further performed a time-dependent growth experiment to evaluate the domain growth speed. Figure 2a–c illustrates the optical images of WS_2_ single crystals at different growth times *t* (*T* = 1300°C). *t* = 0 s is defined as the moment at which the flow direction is switched on reaching the designated growth temperature. At time *t*, the growth was quenched by shutting off the chemical supply and rapidly pulling the quartz tube out from the furnace (Supplementary Fig. S2). We found a growth rate of up to 45 μm/s (Fig. 2d), exceeding the highest growth speed previously reported for TMDs (∼26 μm/s) (Supplementary Table S3).

This ultrafast growth of large monolayer single TMD crystals could be attributed to both the enhanced source supply and increased surface migration rate at a higher temperature, which was not achievable in previous studies. A higher source temperature can promote evaporation of source materials, and enable a rapid lateral growth rate. In addition, the substrate temperature also increased with the source temperature. A higher substrate temperature can enhance the atom migration, as described by the migration equation: $D \approx {D_\infty}{\exp} ( - U/{k_{\rm B}}{T})$, where *k*_B_ is the Boltzmann constant and *U* is the migration barrier energy. At higher temperatures, the migration coefficient is larger and adatoms can diffuse over longer distances before attaching to an island. Recent theoretical calculations showed that the migration energies of W and S atoms on the edge site of WS_2_ are about 3.81 eV and 1.02 eV, respectively [53]. Thus, the lateral growth of WS_2_ is mainly limited by W migration with a migration barrier of 3.81 eV. According to the migration equation, when the substrate temperature increased from 753°C to 875°C (the source temperature increased from 1150°C to 1300°C), the migration coefficient at 875°C will be ∼82 times larger than that at 753°C. This significantly accelerated atom migration at higher temperature can enable rapid lateral growth of monolayer single crystals.

**Figure 3.**
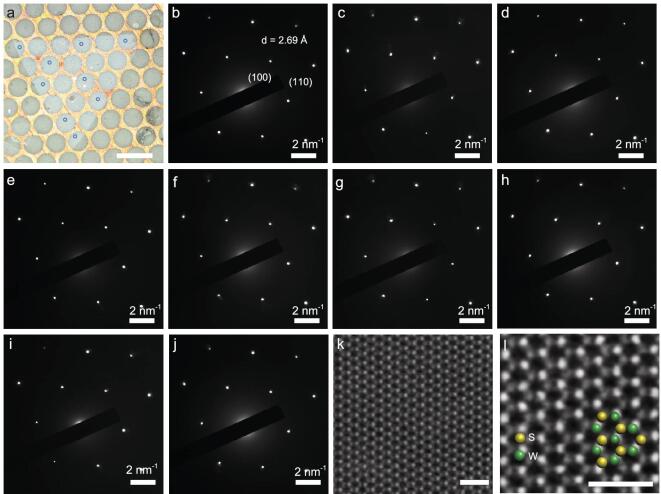
TEM study of monolayer WS_2_ single crystals. (a) OM image of a 900-μm WS_2_ crystal transferred onto a TEM grid. The scale bar is 200 μm. (b–j) Nine SAED patterns from the areas labeled in (a). (k, l) STEM images of WS_2_ crystals. Scale bars are 1 nm.

To evaluate the quality of the large single crystals obtained with the ultrafast growth method, we further investigated the microstructure of the resulting monolayer WS_2_ single crystals using selected area electron diffraction (SAED) and high-angle annular dark-field scanning transmission electron microscope (STEM). Figure 3a shows an OM image of a single monolayer WS_2_ domain (∼900 μm) transferred onto a transmission electron microscope (TEM) grid. The SAED patterns acquired from nine representative areas (as labeled in Fig. 3a) show a single set of hexagonally arranged diffraction spots with identical orientations (Fig. 3b–j), suggesting a single-crystalline lattice structure throughout the entire WS_2_ domain. The atomically resolved STEM image (Fig. 3k, l), with the brighter/dimmer areas corresponding to W/S atoms, shows that the WS_2_ domain exhibits an almost perfect lattice structure with no obvious vacancies and topological defects, confirming the high crystalline quality of the WS_2_ samples.

**Figure 4.**
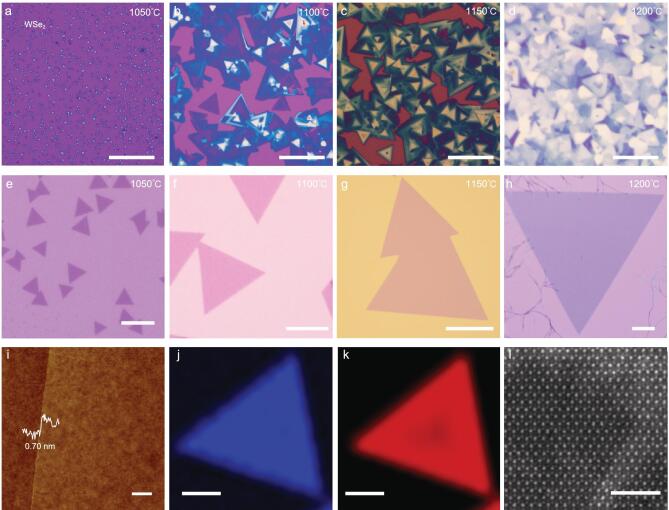
Ultrafast growth of monolayer WSe_2_ single crystals. (a–h) OM images of WSe_2_ grown with a conventional CVD process (a–d) and a modified CVD process with reverse flow (e–h) kept at different temperatures for 10 s. (i) AFM image and thickness of the WSe_2_ single crystal. (j, k) Raman (j) and PL (k) spectroscopy maps of the peaks at 250 cm^−1^ and 760 nm of the monolayer WSe_2_ domain. (l) STEM image of a monolayer WSe_2_ crystal. Scale bars: 10 μm in (e), 100 μm in (a–d, f–h, j, k), 2 nm in (i).

To assess the generality of the reverse flow approach for growth of monolayer single crystals of other TMDs, we also applied this approach to grow monolayer WSe_2_ single crystals. Similarly, a conventional CVD process produces highly heterogeneous thin films at a high temperature (Fig. 4a–d), whereas the modified CVD method with reverse flow produces highly uniform monolayer single crystals (Fig. 4e–h), with increasingly larger WSe_2_ domains obtained at higher temperature. The size of the WSe_2_ crystals can reach 800 μm (Fig. 4f), with the highest growth rate being ∼20 μm/s (Supplementary Fig. S3). An AFM image shows that the thickness of the resulting WSe_2_ single crystal is around 0.70 nm (Fig. 4i). Raman spectroscopic studies show a single prominent peak at 250 cm^−1^, corresponding to the A_1_^′^ resonance mode of monolayer WSe_2_ (Supplementary Fig. S4a), and the PL spectrum shows a single peak at 760 nm (Supplementary Fig. S4b), consistent with the direct bandgap emission of monolayer WSe_2_. The corresponding Raman and PL maps of the monolayer WSe_2_ single crystal domain show a highly uniform contrast (Fig. 4j, k), indicating the high crystalline homogeneity of the monolayer WSe_2_ domain. Furthermore, the STEM image confirms an almost perfect lattice structure (Fig. 4l).

**Figure 5.**
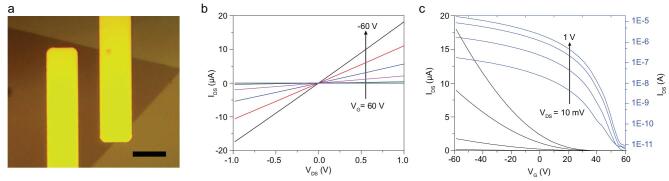
Electronic properties of the monolayer WSe_2_ prepared by the ultrafast growth. (a) OM image of a monolayer WSe_2_ transistor with two transferred Pt electrodes on Si/SiO_2_ substrate; the scale bar is 10 μm. (b) I_DS_−V_DS_ output characteristics of a typical WSe_2_ transistor. (c) I_DS_−V_G_ transfer characteristics at V_DS_ = 10 mV, 100 mV, 500 mV and 1 V. The black and blue curves are the linear plot and semi-log plot, respectively.

To investigate the electrical properties of the resulting monolayer crystals, back-gate field-effect transistors (FETs) were fabricated on SiO_2_/Si substrates with transferred Pt contact electrodes (Fig. 5a) [54]. The I_DS_-V_DS_ characteristics of a typical monolayer WSe_2_ FET show linear and symmetric curves (Fig. 5b), suggesting Ohmic-like contacts. Transfer curves at different V_G_ show that the I_DS_ value decreases monotonically with increasing V_G_ (Fig. 5c), indicating p-type behavior. The measured on/off ratio reaches 2 × 10^6^ at a source-drain bias of 1 V. The carrier mobility can be calculated from the linear regime of the transfer characteristics. Notably, the highest mobility of 90 cm^2^ V^−1^ s^−1^ was

achieved in monolayer WSe_2_ FETs, which is comparable to the best values reported previously for exfoliated monolayer materials and confirms the high crystalline quality of our WSe_2_ crystals [55].

## CONCLUSION

In conclusion, we have developed a modified CVD process using reverse flow during the temperature ramping stage to prevent the unintended supply of chemical vapor source and uncontrolled nucleation and growth, thus greatly enhancing the controllability of the chemical vapor supply and enabling controlled nucleation and rapid growth of millimeter-size-scale monolayer single crystals. Optical and STEM studies reveal the excellent crystalline quality of the resulting 2D crystals. Electrical transport studies further demonstrate that the 2D crystals exhibit excellent electronic characteristics. Thus, our study defines a robust approach to high-quality large-sized 2D single crystals, which is essential for future application of 2D materials in integrated electronics and optoelectronics.

## Supplementary Material

nwz223_Supplemental_File
