# Corrigendum to Ultrafast growth of large single crystals of monolayer WS_2_ and WSe_2_

**DOI:** 10.1093/nsr/nwaa143

**Published:** 2020-08-17

**Authors:** Zhengwei Zhang, Peng Chen, Xiangdong Yang, Yuan Liu, Huifang Ma, Jia Li, Bei Zhao, Jun Luo, Xidong Duan, Xiangfeng Duan

**Affiliations:** State Key Laboratory for Chemo/Biosensing and Chemometrics, College of Chemistry and Chemical Engineering, Hunan University, Changsha 410082, China; State Key Laboratory for Chemo/Biosensing and Chemometrics, College of Chemistry and Chemical Engineering, Hunan University, Changsha 410082, China; State Key Laboratory for Chemo/Biosensing and Chemometrics, College of Chemistry and Chemical Engineering, Hunan University, Changsha 410082, China; Department of Applied Physics, School of Physics and Electronics, Hunan University, Changsha 410082, China; State Key Laboratory for Chemo/Biosensing and Chemometrics, College of Chemistry and Chemical Engineering, Hunan University, Changsha 410082, China; State Key Laboratory for Chemo/Biosensing and Chemometrics, College of Chemistry and Chemical Engineering, Hunan University, Changsha 410082, China; State Key Laboratory for Chemo/Biosensing and Chemometrics, College of Chemistry and Chemical Engineering, Hunan University, Changsha 410082, China; Center for Electron Microscopy, Institute for New Energy Materials and Low-Carbon Technologies, School of Materials, Tianjin University of Technology, Tianjin 300384, China; State Key Laboratory for Chemo/Biosensing and Chemometrics, College of Chemistry and Chemical Engineering, Hunan University, Changsha 410082, China; Department of Chemistry and Biochemistry, University of California, Los Angeles, CA 90095, USA

Zhengwei Zhang and Peng Chen contributed equally to ‘Ultrafast growth of large single crystals of monolayer WS_2_ and WSe_2_’ (*National Science Review*, Volume 7, Issue 4, 2020, Pages 737–744, doi: 10.1093/nsr/nwz223). In the original published version of this manuscript, only Zhengwei Zhang was listed as first author. At the request of the authors, the published manuscript has now been corrected to also include Peng Chen as a first author.

