# Supplementary material for: Ultrafast growth of large single crystals of monolayer WS2 and WSe2
Source: Natl Sci Rev. 2020 Jan 8;7(4):737–44. doi: 10.1093/nsr/nwz223 (PMC8288871; doi:10.1093/nsr/nwz223)
Supplement: nwz223_Supplemental_File [file nwz223_supplemental_file.doc]

**Ultrafast Growth of Large Single Crystals of Monolayer WS2 and WSe2**

Zhengwei Zhang,1† Peng Chen,1,4†  Xiangdong Yang,1 Yuan Liu,2 Huifang Ma,1 Jia Li,1 Bei Zhao,1 Jun Luo,3 Xidong Duan,1* Xiangfeng Duan4

1State Key Laboratory for Chemo/Biosensing and Chemometrics, College of Chemistry and Chemical Engineering, Hunan University, Changsha 410082, China;

2Department of Applied Physics, School of Physics and Electronics, Hunan University, Changsha 410082, China;

3Center for Electron Microscopy, Institute for New Energy Materials and Low-Carbon Technologies, School of Materials, Tianjin University of Technology, Tianjin 300384, China;

4Department of Chemistry and Biochemistry, University of California, Los Angeles, California 90095, USA

E-mail: *xidongduan@hnu.edu.cn*

**Experiment details.**

**Figure S1. Schematic illustration of the reverse flow reactor**

**Figure S2. Schematic illustration of reaction quenching**

**Figure S3. WSe2 growth rate**

**Figure S4. Raman and PL spectrum of WSe2**

**Figure S5. The electrical properties of WS2 monolayer crystals**

**Table S1. Growth parameters for various materials**

**Table S2. Substrate temperature at different source temperature**

**Table S3. Growth rate comparison**

**Experiment details**

**Reverse flow CVD:** To prepare the WS2 (WSe2) monolayer single crystals, an alumina boat loaded with WS2 (Alfa Aesar, 99.8%) or WSe2 (Alfa Aesar, 99.98%) powder (3-5g), was placed into the heating zone of a slender quartz tube (inner diameter 20 mm, length 100 cm). The Si substrates (with 300 nm SiO2) were placed on the alumina boat (Figure S1). Before heating, an Ar flow was introduced into the system at a rate of 300 sccm for 10 min, to ensure a favorable circumstance for the synthesis of the sample. 100 sccm reverse Ar flow is applied from the substrate to source to prevent unintentional chemical vapor supply and avoid nucleation during the ramping up stage (Step 1). Upon reaching and stabilizing at the desired growth temperature, the Ar gas flow direction is switched to forward direction (from the source to substrate), and the heating zone was kept for 10 s to 2 min for the growth of WS2 or WSe2 (Step 2) before the reaction was shut down by switching of the furnace (Step 3). The time plot of the entire growth process is shown in Table S1.

**FET device fabrication process:** We first prepare a series of 50-nm-thick Pt electrodes on a silicon/silicon oxide substrate with an atomically flat surface by using standard photolithography and high-vacuum electron-beam evaporation. Next, a hexamethyldisilazane (HMDS) layer is applied to functionalize the whole wafer, and a PMMA layer is then spin-coated on top of the metal electrodes. With the pre-functionalization by HMDS, the PMMA layer has weak adhesion to the sacrificial substrate and can be mechanically released by using a thermal release tape, together with metal electrodes wrapped underneath. Next, the previously released metal electrodes are aligned under a microscope and physically laminated on top of the WSe2 crystals, resulting in an atomically clean metal-semiconductor interface. Finally, the PMMA on top of the contact pads is removed using standard electron-beam lithography and development processes, leaving the exposed metal pads for electrical probing and measurements.


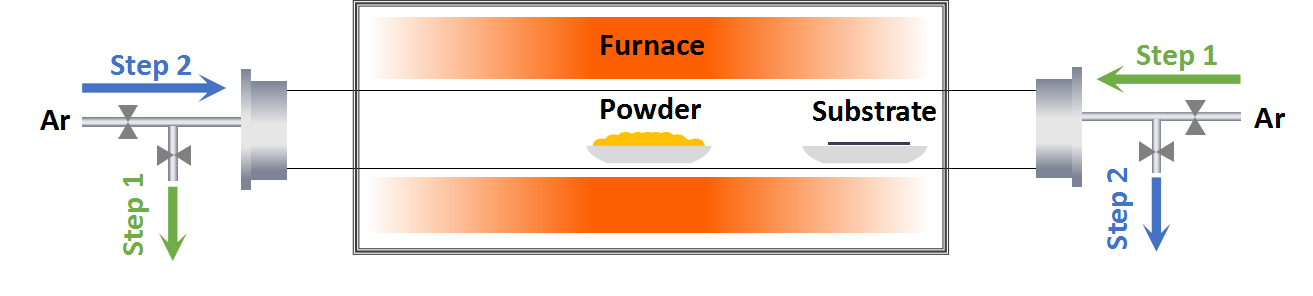


**Figure S1.** Reverse flow reactor. (a) Schematic illustration of a modified CVD system with reverse flow to prevent the unintended chemical vapor supply during the temperature ramping stage for the synthesis of large single crystals of 2D WS2 and WSe2.


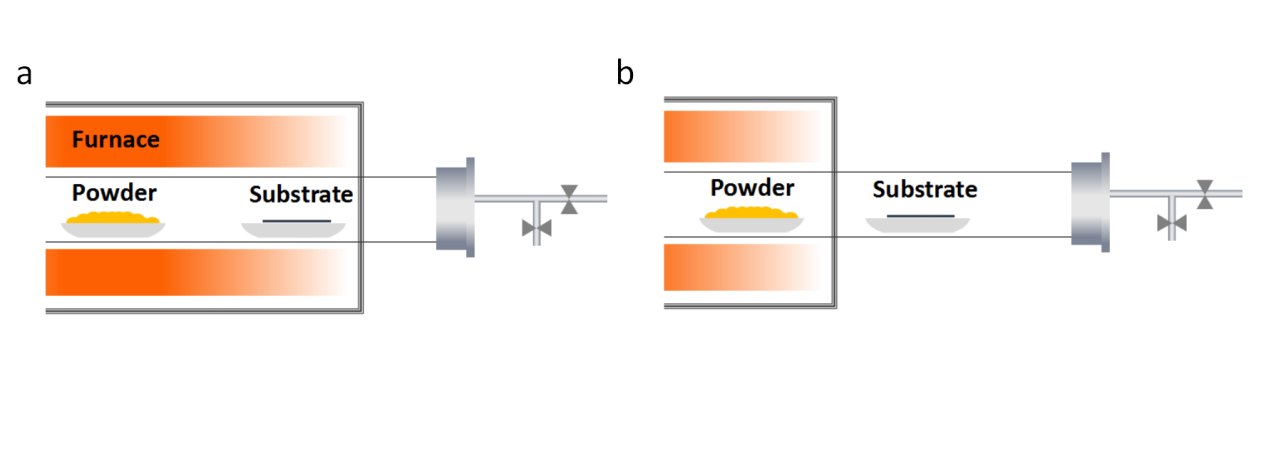


**Figure S2.** The schematic illustration of growth quench method. (a) During the growth. (b) After finish growth, the substrate was pulled out from the furnace immediately to quench the growth.


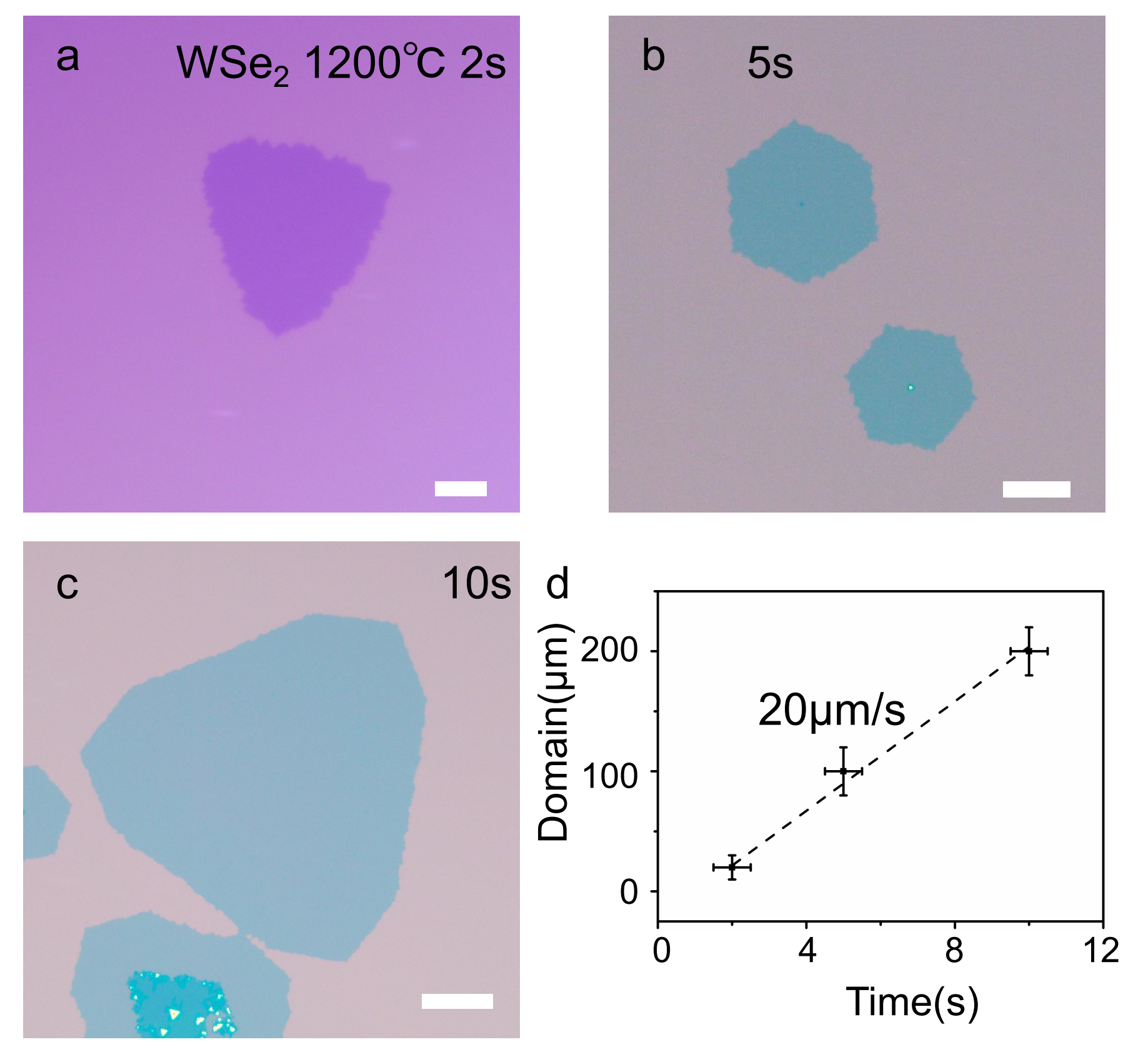


**Figure S3.** Growth rate of WSe2. (a–c), Optical images of WSe2 synthesized at t = 2 s (a), 5 s (b) and 10 s (c), respectively. (d) Plot and fit of WS2 domain size as a function of the growth time. The slope reveals an ultrafast growth rate of ~20 μms-1. Scale bar 10 μm (a), 50 μm (b, c).


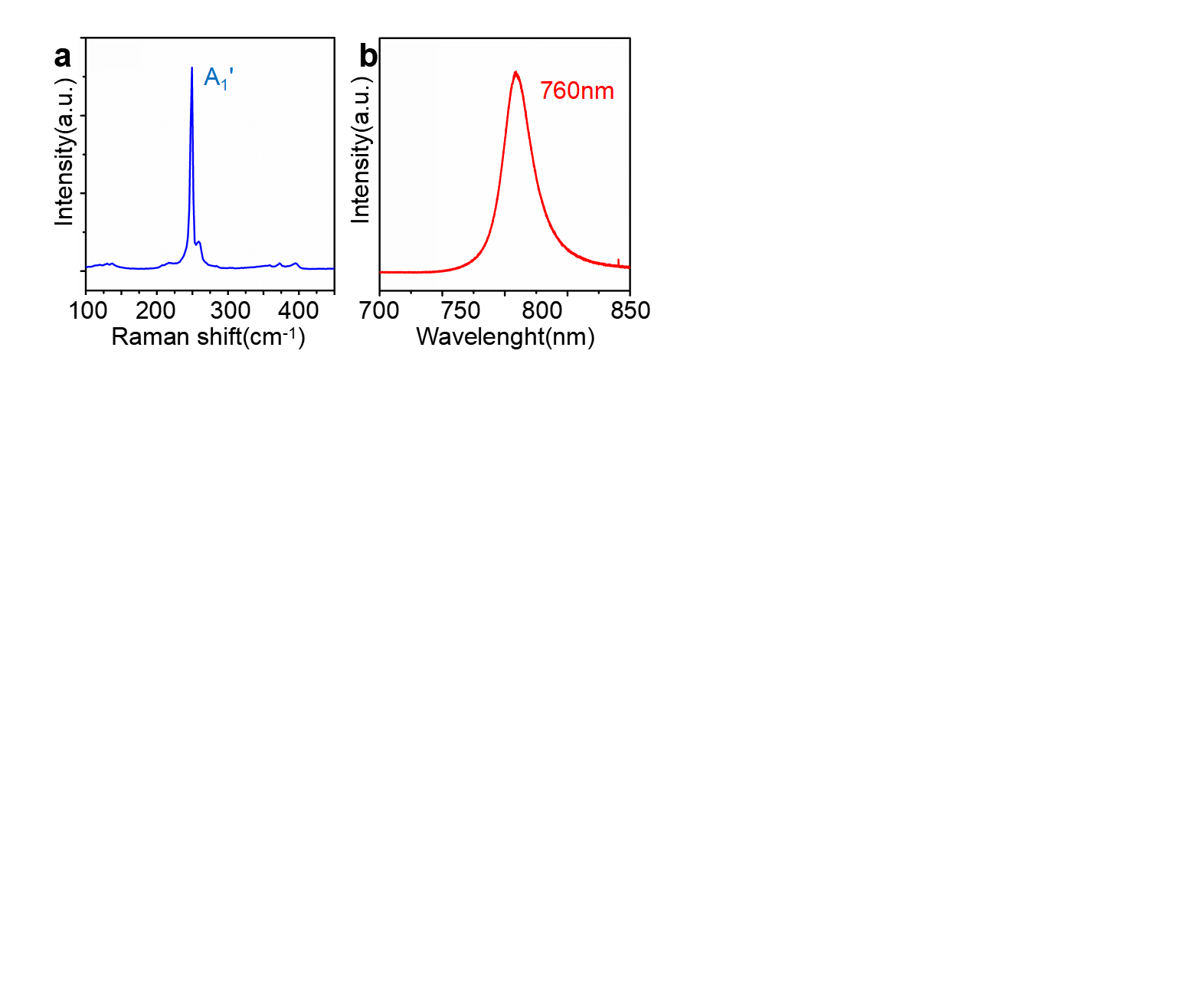


**Figure S4.** The Raman (a) and PL spectra (b) of the monolayer WSe2 domain.


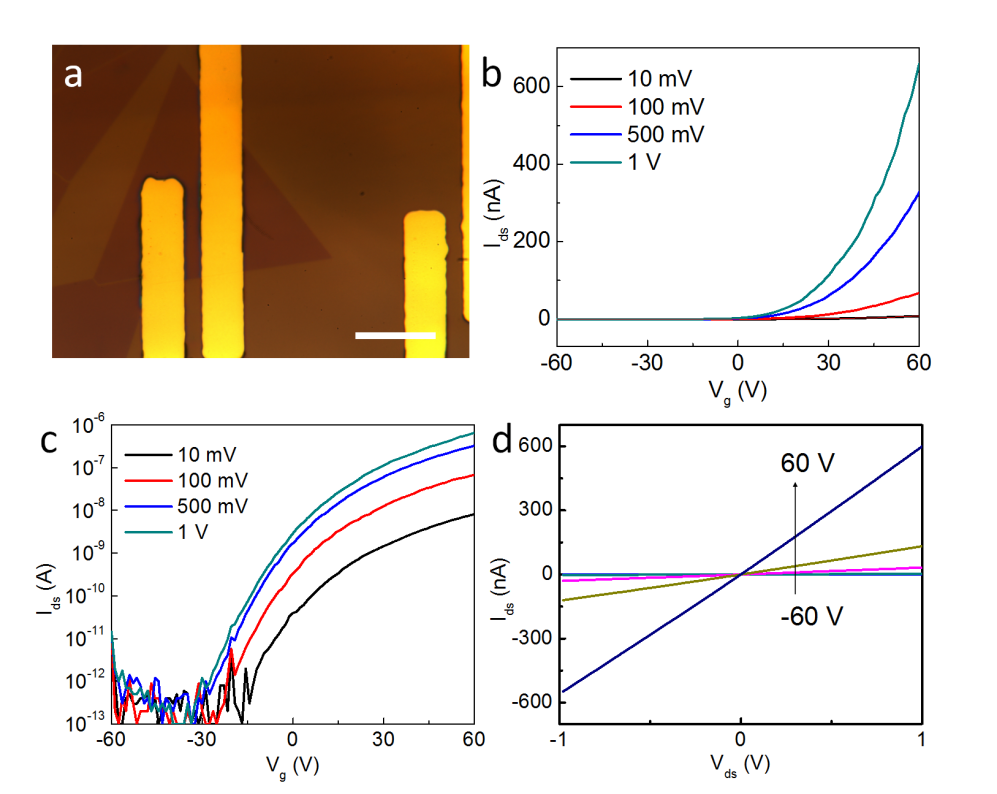


**Figure S5.** The electrical properties of WS­2 monolayer crystals. (a) OM image of a WS2 FET device. (b,c) Linear and logarithmic transfer characteristics of a WS2 FET. (d) Room-temperature output characteristics of the WS2 FET. The carrier mobility is estimated to be ~0.7 cm2V-1s-1, and the ON/OFF current ratio is ~107. which is comparable to the best reported values in CVD WS2.

**Table S1.** Growth parameters for various materials.

|  | Ramping stage | Isothermal stage | | | Max lateral  length (μm) |
| --- | --- | --- | --- | --- | --- |
|  | Reverse Ar flow (sccm) | Forward Ar flow (sccm) | Temp. (℃) | Time (s) |  |
| WS2 | 100 | 90 | 1300 | 10 | ~1000 |
|  | 100 | 90 | 1280 | 10 | ~700 |
|  | 100 | 90 | 1250 | 10 | ~200 |
|  | 100 | 90 | 1220 | 10 | ~75 |
|  | 100 | 90 | 1180 | 10 | ~35 |
| WSe2 | 100 | 100 | 1200 | 10 | ~800 |
|  | 100 | 100 | 1150 | 10 | ~150 |
|  | 100 | 100 | 1100 | 10 | ~20 |
|  | 100 | 100 | 1050 | 10 | ~5 |

**Table S2.** Substrate temperature at different source temperature

| Temperature of the source materials (℃) | Substrate temperature (℃) |
| --- | --- |
| 1100 | 720 |
| 1150 | 753 |
| 1200 | 790 |
| 1250 | 830 |
| 1300 | 875 |

**Table S3.** The comparison of growth rates for CVD growth of TMD single crystals

| GROUP | TMDC materials | Growth rate (μm/s) |
| --- | --- | --- |
| Wencai Ren | WS2 | ~0.069 |
| Guangyu Zhang | MoS2 | ~0.19 |
| Zhongfan Liu | MoS2 | ~0.014 |
| Yanfeng Zhang | MoS2 | ~0.032 |
| Pulickel M. Ajayan | MoSe2 | ~1.30 |
| Kian Ping Loh | MoSe2 | ~8.30 |
| Wencai Ren | WSe2 | ~26 |
| **This work** | **WS2** | **~45** |

1. Lee, Y-H, Yu, L, Wang, H*, et al.* Synthesis and transfer of single-layer transition metal disulfides on diverse surfaces. *Nano letters*. 2013; **13**(4): 1852-7.

2. Gao, Y, Liu, Z, Sun, D-M*, et al.* Large-area synthesis of high-quality and uniform monolayer WS 2 on reusable Au foils. *Nature communications*. 2015; **6**: 8569.

3. Chen, W, Zhao, J, Zhang, J*, et al.* Oxygen-assisted chemical vapor deposition growth of large single-crystal and high-quality monolayer MoS2. *Journal of the American Chemical Society*. 2015; **137**(50): 15632-5.

4. Zhang, Y, Zhang, Y, Ji, Q*, et al.* Controlled growth of high-quality monolayer WS2 layers on sapphire and imaging its grain boundary. *ACS nano*. 2013; **7**(10): 8963-71.

5. Shi, J, Zhang, X, Ma, D*, et al.* Substrate facet effect on the growth of monolayer MoS2 on Au foils. *ACS nano*. 2015; **9**(4): 4017-25.

6. Gong, Y, Ye, G, Lei, S*, et al.* Synthesis of Millimeter‐Scale Transition Metal Dichalcogenides Single Crystals. *Advanced Functional Materials*. 2016; **26**(12): 2009-15.

7. Chen, J, Zhao, X, Tan, SJ*, et al.* Chemical vapor deposition of large-size monolayer MoSe2 crystals on molten glass. *Journal of the American Chemical Society*. 2017; **139**(3): 1073-6.

8. Gao, Y, Hong, YL, Yin, LC*, et al.* Ultrafast Growth of High‐Quality Monolayer WSe2 on Au. *Advanced Materials*. 2017; **29**(29): 1700990.
